# Differences in presentation of symptoms between women and men with intermittent claudication

**DOI:** 10.1186/1471-2261-11-39

**Published:** 2011-06-30

**Authors:** Birgitta Sigvant, Fredrik Lundin, Bo Nilsson, David Bergqvist, Eric Wahlberg

**Affiliations:** 1 Karolinska Institutet, Stockholm, Sweden; 2 Dept. of Surgery, Karlstad Hospital, Sweden; 3 Medical Research Centre, Karlstad Hospital, Sweden; 4 Dept. of Physiology, Karlstad Hospital, Sweden; 5 Dept. of Surgery, Uppsala University Hospital, Sweden; 6 The Heart Centre, Linköping University Hospital, Sweden

## Abstract

**Background:**

More women than men have PAD with exception for the stage intermittent claudication (IC). The purpose of this study was to evaluate differences in disease characteristics between men and women when using current diagnostic criteria for making the diagnosis IC, defined as ABI < 0.9 and walking problems.

**Study Design:**

Cohort study

**Methods:**

5040 elderly (median age 71) subjects participated in a point-prevalence study 2004. They had their ABI measured and filled out questionnaires covering medical history, current medication, PAD symptoms and walking ability. The prevalence of IC was 6.5% for women and 7.2% for men (P = 0.09). A subset of subjects with IC (N = 56) was followed up four years later with the same procedures. They also performed additional tests aiming to determine all factors influencing walking ability.

**Results:**

Men with IC had more concomitant cardiovascular disease and a more profound smoking history than women. Women, on the other hand, reported a lower walking speed (P < 0.01) and more joint problems (P = 0.018). In the follow up cohort ABI, walking ability and amount of atherosclerosis were similar among the sexes, but women more often reported atypical IC symptoms.

**Conclusion:**

Sex differences in the description of IC symptoms may influence diagnosis even if objective features of PAD are similar. This may influence accuracy of prevalence estimates and selection to treatment.

## Background

Peripheral arterial disease (PAD) is common in western countries, affecting almost 20% of elderly populations and its prevalence differs between sexes. Findings of the Swedish PAD Prevalence Study (SPPS) revealed that women are more likely to suffer from PAD when diagnosis is based only on ankle brachial indices (ABI) but when diagnosis relies on additional assessment of symptoms (ie IC) sex differences disappears [1,2].

The prevalence of IC is around 7% among elderly [1], and considerably diminishes patients' quality of life (QoL) [3,4] The extent of this influence on QoL determines whether surgical intervention is indicated. Accordingly, a correct diagnosis, accurate assessment of disease severity and weighing the risk associated with the procedure against the magnitude of potential symptomatic improvement is essential for recommending the best treatment option. A correct diagnosis is also essential to enable appropriate modification of cardiovascular (CV) risk factors and to prevent CV morbidity and death [5].

There are some reports in the literature that men and women with IC are treated differently [6,7]. One example is the amount of resources spent on treatment. According to the Swedish Vascular Registry men undergo more interventions for IC than women [8]. Another example is that women appear to seek treatment when they have more advanced disease than men [6]. It is possible that a sex difference in prevalence is the explanation for this, but patients' perception of their symptoms may also contribute to these observations. The latter is further supported by comparable data for ischemic heart disease [9,10]. Accordingly, any occurring sex difference in IC disease characteristics that may influence making the diagnosis needs to be clarified, since it may influence epidemiologic data and selection to treatment.

We hypothesized that there might be differences in perception of IC symptoms, risk factor occurrence and extent of leg artery atherosclerosis between men and women that may influence diagnosis. The purpose of this study was to evaluate differences in disease characteristics between men and women when using current diagnostic criteria for making the diagnosis IC.

## Methods

The study consists of two parts: A) an analysis of sex differences in risk factors among IC patients in a large cohort of Swedish elderly habitants, and B) an in-depth analysis of IC disease characteristics in a smaller sample from this cohort.

The main purpose of Part A cohort was to estimate general sex differences in perception of walking ability in elderly with and without IC using the SPPS. Briefly, this population based point-prevalence study was conducted in four Swedish regions during 2004 and included questionnaire results of 5080 men and women taken from 8000 randomly selected participants aged 60-90 years. They also had their ABI measured. In this cohort 6.8% (CI 6.5-7.1) had IC. [1]. Questionnaire data collected included details in walking ability, self reported concomitant diseases, medication use, and risk factor occurrence. A detailed description of study design, methodology and validity of SPPS has been published previously [7]. For Part B all subjects with IC living in one of the region (Karlstad) were invited to a follow up study. The purpose of Part B was to elucidate any sex differences in leg artery atherosclerosis distribution, walking ability, perception of symptoms, as well as detecting if any severe heart failure was contributing to a possibly observed sex difference in walking ability

### Study populations

Part A uses the following cohorts from SPPS:

*Group 1. IC*; All subjects with ABI < 0.9 and a positive answer in Rose questionnaire [11], (N = 333, 180 women and 153 men).

*Group 2. Control group; *All subjects with ABI > 0.9 and a negative answer in Rose questionnaire (N = 3313, 1734 women and 1579 men).

A subgroup from Group 1 was used for Part B:

*Group 3. Subgroup IC; *All subjects from Group 1 living in one of the regions used 2004 (Karlstad, N = 88, 51 women and 37 men) screened for possible enrolment in the follow-up study. Nineteen had died in 2008 (nine of CV causes, five defined as non-CV and for five subjects data was missing) the remaining 69 subjects were invited. Of those 81% (n = 56) of the survivors, 35 women agreed to participate. Ten of those subjects were assessed to a home visit because of immobility and only answered the questionnaires and had ABI measured.

### Analyses

#### Part A

Walking Impairment Questionnaire (WIQ) answers, ABI values, Rose questionnaire answers, smoking habits, medication use and concomitant diseases were analyzed for Groups 1 and 2 using data collected 2004.

Concomitant diseases, medication and smoking use were self-reported by the subjects. More details about its use and analysis in presented in a previous publication from SPPS [1].

WIQ is divided in three components estimating walking distance, walking speed and stair-climbing ability [12]. For each component subjects rank the degree of difficulties on a scale from 0 to 4 (0 = unable, 4 = no difficulties) and a summary score is then calculated for each component as a percentage (% score = individual score/4 × 100).

ABI was defined as the ratio of the lowest systolic pressure of the two measured arteries in the ankle divided by systolic blood pressure in the arm [13].

#### Part B

Collected background data consisted of the following

• Medical history (risk factors, co-morbidities and medication use as in Part A),

• Death rate and cause of death (obtained from hospital records),

• ABI (as in Group A).

Part B also covered additional tests to further assess walking ability and QoL:

• Six-minutes walking test (6MWT) [14], which assesses peak walking distance. It took place indoors in a 50 m long hallway with marks every 5 m. Subjects were asked to cover as many laps as possible in 6 minutes. If the subject needed to stop for any reason he or she was allowed to rest but encouraged to resume walking. The clock continued to run during the resting period. The time to the first stop, the cause of the stop and the total distance were recorded.

• Intermittent Claudication Questionnaire (ICQ) is a disease specific instrument for evaluation of QoL. It consists of 16 questions, four evaluating walking distance and stair climbing ability. The results obtained from these questions are presented in a six-graded scale (from totally limited to no limitation at all). The remaining 12 questions correlate with the questionnaires EuroQoL and SF-36 and evaluate daily routines, including physical activities, social activities and physical health, and is scored in a five graded scale [15]. Subjects' responses to each question are summed up and transformed to a 0 to 100 scale, where 0 (the opposite of the WIQ system) is best possible and 100 the worst possible health state.

Examinations in this group also aimed to describe atherosclerotic disease distribution and evaluate effects of heart disease on walking ability:

• Duplex ultrasound (DUS) scanning of leg arteries covered the arterial segments from aortic bifurcation to the Popliteal artery below the knee.

• Echocardiography was performed and as an index of left ventricular systolic function, ejection fraction (EF) was calculated using the apical biplane Simpson's method of discs [16].

Details of these methods and their evaluation is provided at the end of this manuscript as an appendix (Additional file 1).

A left ventricular ejection fraction (EF) less than 50% was considered as an index of impaired systolic function.

Part B also included a descriptive analysis of symptoms using a semi-structured interview. It was conducted by asking each subject eight questions with open answering alternatives (Table 1). Questions were designed together with an expert nurse in QoL research. The interview took place in a quiet consulting room and a tape recorder was used.

**Table 1:** Semi-structured Interview Questions

1. Do you have any pain while walking?
2. If, can you describe the symptoms
3. In what way does the symptom influence you?
4. Have you searched medical attention for your problem?
5. How often do you leave your home?
6. What kind of activities do you do?
7. Are you independent in activity in daily life?
8. If you are dependent on help, who will help you?

### Ethical approval

The sub-study was approved by the Ethics committee at Uppsala University (Dnr 2008/056). and SPPS by five separate committees Stockholm (KI 03-538), Umeå University (Dnr 03-459) Lunds University (Dnr832-0) Uppsala University (Dnr 03-564) and Örebro (Dnr 374-03). Informed consent was obtained from each participant.

## Statistics

Binary data was described using numbers and proportions. For age we used sample means and standard deviations. All other data are described by medians and interquartile range (IQR). For ABI we used non-parametric statistics (both descriptive and adjusted by means of non-parametric quartile regression on the median) since it showed significant deviations (Shapiro-Wilks test, p < 0.001) from the normal distribution. Within group differences between sexes was compared using Mann-Whitney U-test. Presented correlations are Spearman rank correlations tested for deviations from the null hypothesis using an asymptotic test. For comparing proportions between sexes we used Fisher's exact test. All analyses were performed with STATA/IC for Windows version 10 (STATA Corp LP. College Station, Texas). P-values below level 0.05 were considered to be statistically significant.

## Results

### Part A

Baseline data for Group 1(IC patients) (n = 333) and Group 2 (control subjects) (n = 3322) are presented in Table 2. Group 1 subjects were older compared to Group 2 (p < 0.001). Men were more likely to have suffered a stroke in both groups (Group 1 p < 0.001; Group 2 p = 0.007). In Group 1 men were more likely to suffer from DM than women (p = 0.047), and in both groups men were more likely to have CAD than women (Group 1 men p < 0.001; Group 2 p < 0.001). Smoking habits, while more common in Group 1 compared to Group 2 (62.5% vs 50.8%, p < 0.001), revealed similar patterns from a sex perspective in both groups. There was however an increased likelihood of a smoking history in men (Group 1 p < 0.001; Group 2 p < 0.001).

**Table 2:** Baseline characteristics

	Group 1(IC)		Group 2 (Control)		Group 3(Follow-up IC)	
	Men (N = 153) (46.0%)	Women (N = 180) (54.0%)	Men (N = 1579) (47.7%)	Women (N = 1734) (52.3%)	Men (N = 37) (42.1%)	Women (N = 51) (58.0%)
**Age** Mean (Std.dev.)	76.3(8.1)	77.0(7.8)	69.9(7.3)	70.6(7.6)	78.7(8.12)	77.5(7.96)
**ABI** Median (IQR)	0.72(0.19)	0.71(0.29)	1.07(0.14)	1.00(0.10)	0.69 (0.21)	0.71 (0.29)
(p-value)***	(0.438)		(< .001)		(0.74)	
**Concomittant diseases** **N (%)**						
Diabetes n = 3646	42 (27.5)	32 (17.8)	125 (7.9)	119 (6.9)	9 (25.0)	4 (7.7)
(p-value)**	(0.047)		(.258)		(0.033)	
Hypertension n = 3646	86 (56.2)	97 (53.9)	494(31.3)	592(34.1)	19 (52.8)	31 (59.6)
(p-value)**	(0.740)		(0.082)		(0.662)	
Stroke n = 3646	38 (24.8)	12 (6.7)	103 (6.5)	76 (4.4)	9 (25.0)	3 (5.8)
(p-value)**	(< 0.001)		(0.007)		(0.013)	
CAD# n = 3646	26 (17.0)	8 (4.4)	271 (17.2)	222 (12.8)	6 (16.7)	3 (5.8)
(p-value)**	(< 0.001)		(< 0.001)		(0.151)	
**Medication use** **N (%)**						
Anti-platelet therapy, n = 2380	134 (99.3)	151 (96.8)	868 (91.5)	1066 (93.5)	34 (100)	47 (97.9)
(p-value)**	(0.221)		(0.079)		(1.00)	
Lipidlowering therapy, n = 2380	53 (39.3)	45 (28.9)	238 (25.1)	214 (18.8)	12 (35.3)	13 (27.1)
(p-value)**	(0.064)		(< 0.001)		(0.472)	
ACE-inhib. Betablockers n = 2380	73 (54.7)	82 (52.6)	420 (44.3)	397 (34.8)	25 (73.5)	28 (58.3)
(p-value)**	(0.815)		(< 0.001)		(0.170)	
**WIQ subscales**						
WIQ distance*	44.6	36.1	100.0	100.0	34.5	40.3
(p-value)***	(0.121)				(0.833)	
WIQ speed*	26.1	21.7	89.1	68.5	20.7	23.9
(p-value)***	(0.013)		(< 0.001)		(0.860)	
WIQ stairs*	43.8	41.7	100.0	87.5	41.7	37.5
(p-value)***	(0.13)		(< 0.001)		(0.937)	
**Smoking ever** **N (%)**						
Yes	119 (77.8)	89 (49.4)	968 (61.3)	716 (41.3)	26 (72.2)	29 (55.8)
(p-value) ***	(< 0.001)		(< 0.001)		(0.179)	

Walking distance (p < 0.001) and speed (p < 0.001) as well as stair climbing ability (p < 0.001) were significantly worse for women as compared to men in Group 1. While women in Group 2, experienced more problems with lower walking speed (p = 0.013), joint problems (p = 0.018) and heart palpitations (p = 0.002).

### Part B

Women and men in Group 3 decreased ABI values between 2004 and 2008 to a similar extent, from 0.67 (SD 0.13) to 0.44 (0.39) in men and 0.69 (0.15) to 0.44 (0.40) in women (p > 0.001). Walking ability as assessed by WIQ also changed during the four years in all domains for men who reported declined scores for pain 78.6(SD 19.8) to 66.2 (30.6), distance from 54.2 (36.4) to 35.4 (30.6) and for speed from 34.9 (22.9) to 20.1 (20.2). The corresponding figures for women were for pain, distance- and speed respectively; 65.7 (33.8) to 76.4 (33.2), 47.3 (35.8) to 49.3 (41.7) and 31.5 (25.8) to 21.8 (14.6).

Walking distances were similar between the sexes, with pain free distance of 111 m (SD 132) for men and 137 m (SD 160) for women (P = 0.58), and absolute distances of 288 m (SD 138) for men and 296 m (SD 160) for women (P = 0.19).

**Figure 1:**
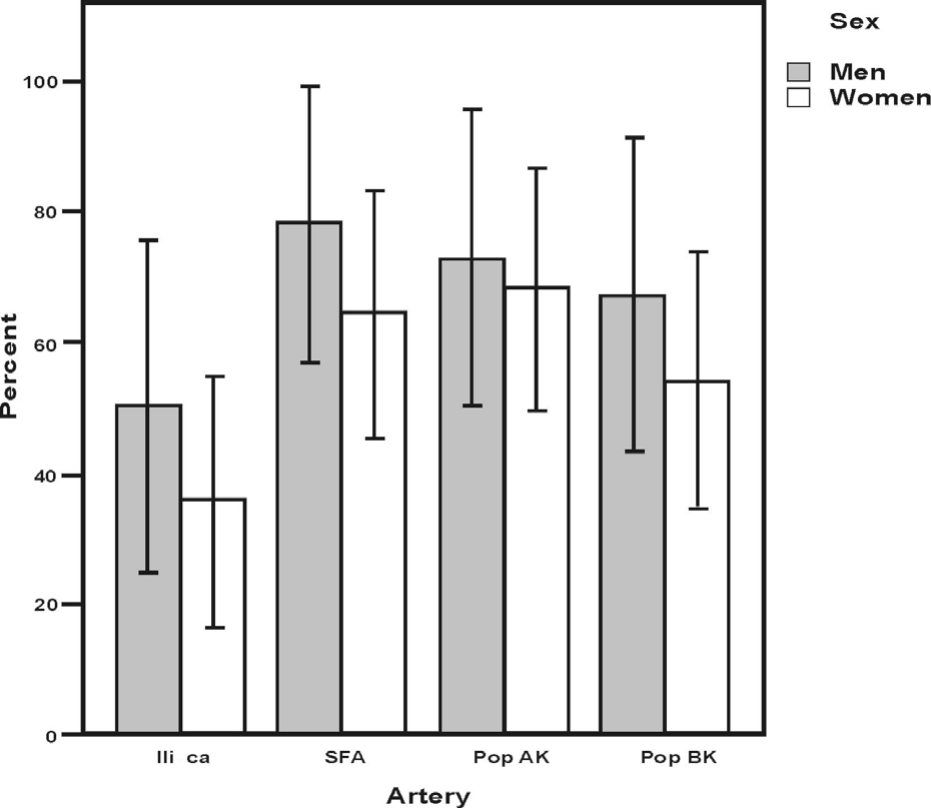
**Frequencies of stenosis and/or occlusions in lower limb arteries imaged by Duplex Ultrasound separated by sex in subgroup IC (with 95% CI) **(Iliaca corresponds to the Common and External Iliac Artery, SFA to the Femoral Superficial Artery, PoP AK and BK to the Popliteal Artery above and below knee).

ICQ data showed that women spent more time thinking about leg pain (p = 0.045) and although not statistically significant showed that men were more likely to report severe leg pain, (p = 0.067) (Table 3). There was no significant difference in the presence of vessel stenosis identified on DUS between men and women at the level of the Iliac artery (p = 0.373), Superficial Femoral artery (p = 0.513), and or Politeal artery (p = 1.0) (Figure 1). For the heart. left ventricular systolic dysfunction tended to be more common in men, with a reduced ejection fraction in 41% of men compared to 15% of women (P = 0.06). No significant correlation was observed between EF and 6MWT distances (rho = 0.21, p = 0.19).

**Table 3:** Responses of the Intermittent Claudication Questoinnaire in Group 3 (Subgroup IC)

*Score Median and Quartiles (Q)*	Men			Women			P-value*
	Q1	Median		Q1	Median	Q3	
Severity of leg pain	3	3	4	0.75	3	4	0.067
Pain limiting using bus, train or tube	0	0	2	0	0	2	0.992
Pain limiting climbing several flights of stairs	0	0	0	0	1	2	0.346
Pain limiting climbing one flight of stairs	2	3	3	1	2	4	0.319
Pain limiting walking more than 1 mile	1	1	2	0	1	3	0.551
Pain limiting walking 100 yards	2.5	3	4	1	4	4	0.934
Pain limiting leaving house	0	1	2	0	0	2	0.827
Pain when stopped walking	0	1	3	0	1	3	0.972
Time spent thinking about leg pain	0	1	3	0	2	3	**0.045**
Felt downhearted and low because of pain	2	3	3	1	2	3	0.075
Time spent worrying that pain will worsen	0	1	2.5	0	1	3	0.640
Interference with normal work	1	2	2.5	0	1	2	0.162

(0 = best possible health state, 4 = worst possible health state)

* P-values comparing differences between sexes using Mann-Whitney's U-test.

Men presented classic IC symptoms (i.e. pain and cramp) in the interview (Table 1 question 2) more frequently than women who in turn more often described symptoms as tiredness, unsteadiness, numbness and sorrow (75% vs. 41% p = 0.086). A higher proportion of men regularly left their homes (25% vs 7%, p = 0.097), and men sought medical care three times as often as women (67% vs 19% p = 0.011). Sixty-three percent of the interviewed men (and 52% of women) were dependent on aid and a majority of them was supported by their spouses (89%), whereas women used professional assistance (57%) or their children (36%).

## Discussion

This study presents some new findings that may be of importance when diagnosing and evaluating IC. First, men are more likely to have suffered from a CV disease than women and women tend to perceive a reduced walking ability more often than men do regardless if they are diagnosed as suffering from IC or not. Second, women and men diagnosed with IC appear to have similar extent and type of disease when objective measures are employed. Third, when these IC patients are interviewed about their problems, women more often describe a reduced need of walking ability, or habit of walking compared to men.

Men have a greater CV disease burden than women in the present study, which is a common finding throughout the current published literature. It is valid also after adjustment for age [17,18] and supports the notion that IC should be more common in men [2,19-21]. IC was indeed more common in men in the few early IC prevalence studies that enrolled women [20,22], but more recent publications report a sex neutral prevalence [23-25]. The reasons for a diverging pattern in prevalence for IC compared to other PAD stages could be explained by most epidemiological studies use of only questionnaire as diagnostic tool for IC diagnosis [26]. In Part A of this study that combined questionnaires with ABI for IC diagnosis it appears that the IC patients are correctly diagnosed. This is supported by Duplex findings of leg artery in the follow up study. Accordingly, falsely diagnosed IC patients in this study were not identified.

While the specificity of Rose Questionnaire and an ABI criteria appears to have a good sensitivity for diagnosing IC, this combination may still fail to diagnose some patients with IC. There are a few indications that this may have occurred for women in the present study. Men but not women in the subgroup IC had deteriorated WIQ scores over time. This can be explained if men had correctly diagnosed IC in 2004 that then deteriorated over time at a pace that can be expected for IC patients, while some women had a walking problems caused by a non-vascular disease that doesn't deteriorate at the same rate. One example of such could be spinal stenosis [27]. It is also reported that women with IC ambulate slower than men, which can influence diagnosis [28]. In our study, on the other hand, there was an identical reduction in ABI and walking distances over time in both sexes with IC. Another possibility is that WIQ is not an accurate instrument for assessing walking ability in women. WIQ is validated against treadmill walking but atypical leg symptoms are not well covered by this instrument [29].

Interestingly, women from the Group 2 (control) scored lower in all WIQ domains, and thus experienced more leg problems, despite men reporting more concomitant CV disease. In general, women's walking problems appear to be more imprecise and distorted by bodily pain, stiffness in joints, discomfort in the chest and general weakness. It is likely that such general perception will make IC symptoms more indistinct in women, and this may influence prevalence data using Rose Questionnaire. Whether more recently developed instruments such as the Edinburgh Claudication Questionnaire or similar ones will solve this potential problem of under-diagnosing IC in women is unknown [11,30]. Few studies have addressed the problem of atypical IC symptoms in PAD but it is well-documented for coronary heart disease [31-33].

Diagnosis of IC also depends on the degree of walking impairment but all subjects with IC needs to be diagnosed to enable CV risk reduction measures, including very mild IC, that do not requires symptomatic treatment. Whether women and men with IC have different walking abilities is unknown. Gardner et al. reported that men and women with IC had similar 6MWT walking distances, but in a treadmill test women walked shorter distances [34]. The explanation given for this observation was a reduced pulmonary function that influenced walking ability in women. McDermott also found a greater functional impairment among women with IC compared to men. The reason given was diminished leg muscle strength in women [27]. Our data reports a similar disease distribution between the sexes on DUS and a possibly better heart function in women, opposing the notion that women have less severe PAD. Overall it is difficult to find clear support for an appropriate sex difference in resource allocation for symptomatic treatment of IC.

The severity of walking impairment is only one component of the clinical entity IC that is important to consider when deciding whether a patient should be offered symptomatic treatment. A short walking distance might be a severe disability for one person but perfectly acceptable for another. While the only sex-differences in ICQ scores concerned leg pain (where men experienced more problems), women reported lower physical function and ability to perform daily life activities. These results are consistent with a previous data where women perceived a lower physical function, mood state and greater bodily pain than men despite similar disease severity [31,35]. Accordingly, there appears to be a clear sex difference in perception of the limitation caused by IC. This difference may be a consequence of divergent walking habits and needs. In the interviews performed in this study it was obvious that men were more physically active than women and that women did not leave their houses as often and were more dependent of support [24,35,36]. Despite the fact that a compromised walking ability is the main feature of IC disease, only 34% of the subjects in Group 3, who reported that they could walk slightly more than 100 m, had been subject to medical care for their disease, and more men had received treatment. If women tend to ignore the severity of their IC symptoms they do not look for medical care.

Some limitations of this study are apparent. Few statistically significant differences between the sexes were observed in Part B, which could be caused by the small cohort size and it is possible that larger and more significant differences between the sexes would have been identified if a larger cohort had been recruited. Another potential issue is the ability to generalize our results to the entire IC population. Group 3 was an acceptable approximation of the demographics of Group 2 (Table 4), but while criteria used to identify this cohort are applicable for epidemiology studies but not entirely consistent with day to day clinical praxis as for example self reported questionnaires.

**Table 4:** Baseline characteristics for Group 2 and 3

	Group 2 N = 333	Group 2 Except group 3 N = 245	Group 3 N = 88	p-values
Age	76.7 (7.9)	76.2 (7.0)	78.0 (7.9)	0.97
ABI	0.69 (0.16)	0.70 (0.17)	0.68 (0.15)	0.14
Diabetes	74 (22.2%)	61 (24.9%)	13 (14.8%)	0.05
Hypertension	183 (54.9)	133 (54.3)	50 (56.8%)	0.71
Stroke	50 (15.0%)	38 (15.5%)	12 (13.6%)	0.73
CAD	34 (10.2%)	25 (10.2%)	9 (10.2%)	1.00
Smoking	208 (62.5%)	153 (62.5%)	55 (62.5%)	1.00

Differences betwen group are tested by Fisher's exact test except for age and ABI where the t-test Mann-Whiney's U-test was used respectively.

## Conclusion

Sex differences in the description of IC symptoms may influence diagnosis even if objective features of PAD are similar. Men claim to suffer from more pain while women have a greater walking impairment and describe symptoms more atypical. IC prevalence and its consequences may be underestimated in women and this need to be considered in epidemiological studies, clinical trials and as indication for revascularization. Accordingly, the results of this study suggest that atypical symptoms of IC among women should be viewed as having a similar consequence on exercise performance as that of classical IC or similar.

## Abbreviations

PAD: Peripheral Arterial Disease; IC: Intermittent Claudication; ABI: Ankle Brachial index; SPPS: Swedish PAD Prevalence Study; CV: Cardio Vascular; WIQ: Walking Impairment Questionnaire; ICQ: Intermittent Claudication Questionnaire; 6MWT: Six minutes Walking Test; QoL: Quality of Life; DUS: Duplex Ultra Sound; EF: Ejection Fraction.

## Competing interests

E Wahlberg has received independent research grants from. Pfizer AB. and speakers fees from Bayer-Schering, and BMS .F Lundin, B Nisslon D Bergqvist and B Sigvant declare that they have no financial competing interests

## Authors' contributions

BS designed and coordinated the study, participated in the data analysis and writing of the paper. FL preformed the statistical analysis. BN collected data and participated in data analyses and commented the draft. DB participated in the design and commented the draft. EW designed, participated in the data analysis and writing of the paper

All authors read and approved the final manuscript.

## Pre-publication history

The pre-publication history for this paper can be accessed here:

http://www.biomedcentral.com/1471-2261/11/39/prepub

## Supplementary Material

Additional file 1 **Methodology and evaluation of Duplex ultrasound and Echocardiography**. An appendix with detailed description of methodology of preformed Duplex ultrasound of leg arteries and Echocardiography Click here for file
