# Supplementary material for: Differences in presentation of symptoms between women and men with intermittent claudication
Source: BMC Cardiovasc Disord. 2011 Jun 30;11:39. doi: 10.1186/1471-2261-11-39 (PMC3141760; doi:10.1186/1471-2261-11-39)
Supplement: Additional file 1 — Methodology and evaluation of Duplex ultrasound and Echocardiography. An appendix with detailed description of methodology of preformed Duplex ultrasound of leg arteries and Echocardiography [file 1471-2261-11-39-S1.DOC]

**Additional file**

**Additional file 1.**

*Title*: Methodology and evaluation of Duplex ultrasound and Echocardiography

*Description:* An appendix with detailed description of methodology of preformed Duplex ultrasound of leg arteries and Echocardiography

**Duplex ultrasound (DUS)** scanning of leg arteries was carried out using an Acuson Sequoia c 512 (Siemens, US) scanner. An experienced vascular technologist performed the examinations that covered the arterial segments from aortic bifurcation to the popliteal artery below the knee. The degree of stenosis was calculated by using peak systolic velocity (PSV) ratio criteria (ratio of PSV at the diseased segment to that of normal proximal segment). DUS findings were stratified into four categories: 1. Normal (PSV ratio <2.5 m/s), 2. Significant stenosis (PSV >2.5), 3. Diffuse disease (numerous lesions without any PSV alteration or PSV<2.5) and 4. Occlusion (absence of flow) {Lowery, 2007 #175}.

**Echocardiography** was performed using a Vivid 7 system (GE, Vingmed Ultrasound, Horten, Norway) with a 3, 5 MHz phased-array transducer with patients in the left lateral recumbent position. Cine-loops including at least 3 consecutive heartbeats were saved and transmitted to a work-station (EchoPAC-PC version 6.0; GE Medical systems) for off-line analysis. Two and four chamber views were obtained. As an index of left ventricular systolic function, ejection fraction (EF) was calculated using the apical biplane Simpson´s method of discs {Lang, 2005 #251}.
